# Transplantation of canine olfactory ensheathing cells producing chondroitinase ABC promotes chondroitin sulphate proteoglycan digestion and axonal sprouting following spinal cord injury

**DOI:** 10.1371/journal.pone.0188967

**Published:** 2017-12-11

**Authors:** Darren Carwardine, Jonathan Prager, Jacob Neeves, Elizabeth M. Muir, James Uney, Nicolas Granger, Liang-Fong Wong

**Affiliations:** 1 School of Veterinary Sciences, University of Bristol, Bristol, United Kingdom; 2 Department of Physiology Development and Neuroscience, University of Cambridge, Cambridge, United Kingdom; 3 Bristol Medical School, University of Bristol, Bristol, United Kingdom; University of Toronto, CANADA

## Abstract

Olfactory ensheathing cell (OEC) transplantation is a promising strategy for treating spinal cord injury (SCI), as has been demonstrated in experimental SCI models and naturally occurring SCI in dogs. However, the presence of chondroitin sulphate proteoglycans within the extracellular matrix of the glial scar can inhibit efficient axonal repair and limit the therapeutic potential of OECs. Here we have used lentiviral vectors to genetically modify canine OECs to continuously deliver mammalian chondroitinase ABC at the lesion site in order to degrade the inhibitory chondroitin sulphate proteoglycans in a rodent model of spinal cord injury. We demonstrate that these chondroitinase producing canine OECs survived at 4 weeks following transplantation into the spinal cord lesion and effectively digested chondroitin sulphate proteoglycans at the site of injury. There was evidence of sprouting within the corticospinal tract rostral to the lesion and an increase in the number of corticospinal axons caudal to the lesion, suggestive of axonal regeneration. Our results indicate that delivery of the chondroitinase enzyme can be achieved with the genetically modified OECs to increase axon growth following SCI. The combination of these two promising approaches is a potential strategy for promoting neural regeneration following SCI in veterinary practice and human patients.

## Introduction

Trauma to the spinal cord can lead to permanent loss of function. The injury initiates a cascade of secondary biochemical events including inflammation, excitotoxicity, demyelination, axonal loss and cell death, causing irreparable sensory and motor dysfunction [1–3]. As the pathophysiology following spinal cord injury (SCI) is complex, a combination of approaches is likely to be required in order to combat the diverse factors impairing recovery [4–6].

The glial scar is a major contributor to failed regeneration in the mammalian central nervous system due to the inhibitory effect of chondroitin sulphate proteoglycans (CSPGs) on regenerating axons [7]. Enzymatic digestion of the glycosaminoglycan side-chains on CSPGs with the bacterial enzyme chondroitinase ABC (ChABC) leads to axonal regeneration and plasticity [8–10]. In addition, ChABC disrupts the perineuronal nets found around intact synapses leading to increased plasticity in surviving neurons [11, 12]. Together, these mechanisms have resulted in functional improvement in experimental spinal cord injury (SCI) in rodents [13–17] and in experimental spinal cord hemisection in cats [18, 19] following ChABC delivery.

The ChABC enzyme rapidly degrades at body temperature [20] and this has limited its translational potential. To circumvent this, the ChABC protein has been thermo stabilised using a sugar trehalose to prolong its activity for up to 4 weeks [21]. The bacterial *ChABC* gene has also been modified to allow effective secretion from mammalian cells [22] and delivery of this gene into the injured spinal cord leads to large scale digestion of CSPGs *in vivo* [23, 24]. Due to its pleiotropic actions which do not overlap with other experimental therapies, ChABC is thought to work well in combination with other therapies, such as cell transplantation, to encourage recovery from SCI [6].

Cell transplants, such as olfactory ensheathing cells (OECs), can be used as a substrate to support nerve regeneration, addressing the lack of trophic provision neurons encounter following central nervous system injury [25]. Other beneficial effects of OECs include their ability to modulate the immune response [23], interact with reactive astrocytes [26], promote remyelination and form cell bridges to facilitate neural regeneration [27]. OEC transplantation in spinal cord lesions has been shown to improve locomotor function in experimental models of SCI [28, 29], in dogs with naturally occurring injuries [30], and have progressed to clinical trials in people [31–33].

Naturally occurring canine SCI represents a useful translational bridge between experimental rodent models of SCI and human clinical trials [34–36]. Canine OECs can be safely collected from the nasal mucosa, cultured *in vitro* and autologously transplanted into spinal cord lesions [30]. We have previously demonstrated that these canine OECs can be genetically modified to produce the mammalian-modified form of ChABC *in vitro* [37]. We further show here that canine OECs can act as a vehicle for delivering active ChABC to the site of SCI in rats. This novel combination therapy is effective at digesting CSPGs *in vivo*, increasing axonal regeneration and sprouting.

## Materials and methods

All procedures were performed in accordance with the United Kingdom Animals (Scientific Procedures) Act 1986 and were approved by the University of Bristol Animal Welfare and Ethical Review Body.

For procedures, animals were anaesthetised with intraperitoneal injections of 60mg/kg ketamine and 0.25mg/kg medetomidine. Post-operative analgesia was provided in the form of subcutaneous injections of 0.01mg/kg of buprenorphine.

For euthanasia, all animals were deeply anaesthetised using sodium pentobarbital (200mg/kg intraperitoneal) and transcardially perfused with 0.9% saline followed by 4% paraformaldehyde in 0.1M phosphate buffer.

### Cell culture

Olfactory mucosa cells were collected from a fresh canine cadaver within 10 minutes of euthanasia. This pet dog presented to our veterinary clinic and was euthanized for medical reasons unrelated to this research. Owner consent was obtained and ethical permission was granted by our local ethical committee (Veterinary Investigation Number: 13/033). Cells were collected via endoscopic nasal mucosa biopsy and prepared according to previously published methods [38]. Cells were maintained in culture on poly-l-lysine coated flasks containing DMEM, 10% FBS, 2μM forskolin, 20 ng/mL neuregulin-1 and 1% penicillin and streptomycin. All cells in culture were fed by replacing half of the media with fresh media every 3–4 days.

### Lentiviral production and transduction

The lentiviral transfer vector used to deliver the mammalian-compatible *Proteus vulgaris ChABC* gene [22] and the *GFP* gene to canine OECs was identical to the pRRL-CMV-ChABC-SFFV-GFP vector previously reported [37]. The control transfer vector pRRL-CMV-GFP expressed solely GFP. One week prior to cell transplantation canine OECs were passaged into 24 well plates (1.9 cm^2^ diameter wells) at 2 x 10^4^ cells/cm^2^. Cells were transduced at 1 day *in vitro* with the either the lentiviral vector pRRL-CMV-ChABC-SFFV-GFP or pRRL-CMV-GFP at a multiplicity of infection (MOI) of 10.

### Morgan-Elson assay

A Morgan-Elson assay [39] was performed on 6 samples of cell culture media from each group immediately prior to cell transplantation. This assay detects the disaccharide breakdown products of CSPG digestion following exposure to ChABC. N-acetylation of the disaccharide degradation products results in a colour change. This assay has been adapted to allow a quantitative measure of active ChABC enzyme. Concentrations are expressed in U/mL [22] and performed as described in [37].

### Immunocytochemistry

Cells were fixed with 4% paraformaldehyde for 10 min and immunolabelled using mouse anti-nerve growth factor receptor (p75^NGFR^) (MAB5264, Millipore, Germany; 1:200), rabbit anti-fibronectin (A0245, Dako, Denmark; 1:400) and chicken anti-GFP (Abcam; 1:2000). Secondary antibodies were anti-mouse 546 (Abcam, UK; 1:500), anti-rabbit 660 (Abcam, UK; 1:400) and anti-chicken 488 (Abcam, UK; 1:500). Coverslips were mounted using hard-set mounting medium containing DAPI (Vectashield). The transduction efficiency was calculated using ImageJ by counting the number of p75^NGFR^ positive cells (OEC marker) that were also GFP positive. Cell purity was calculated by counting the number of p75^NGFR^ positive cells and the number of fibronectin positive cells (olfactory nerve fibroblasts) and expressing this count as a percentage of the total number of DAPI positive cells.

### Spinal cord injury and cell transplantation

All procedures were performed in accordance with the United Kingdom Animals (Scientific Procedures) Act 1986 and were reviewed by the University of Bristol Ethical Review Group. Experiments were performed on adult 250g male athymic nude rats (ENVIGO, hsd:RH-*Foxn1*^rnu^), housed with an enriched environment under a standard 12 h light/dark cycle, in a laminar flow unit, with *ad libitum* access to food and water. Nude rats were used to reduce the risk of rejection following xenotransplantation. All animals were anaesthetised with intraperitoneal injections of 60mg/kg ketamine and 0.25mg/kg medetomidine. The skin over the dorsal cervical region was clipped and aseptically prepared. Following a dorsal laminectomy at the 4^th^ cervical vertebrae, the dura was cut to expose the spinal cord in this region. A dorsal column crush to damage the corticospinal (CST) tract was performed as previously described [13]. Post-operative analgesia was provided in the form of subcutaneous injections of 0.01mg/kg of buprenorphine every 6 hours.

Cell cultures were prepared for transplantation on the day of surgery and injected into the spinal cord within 1 hour of detachment from flasks. Cells were trypsinised, washed twice in calcium and magnesium free Hank’s balanced salt solution and resuspended in the same solution at a cell concentration of 4 x10^4^ cells per μL. A total of 8 x10^4^ cells were injected into the spinal cord with two injections of 4 x10^4^ cells each. Injections were given immediately rostral and caudal to the lesion, at a depth of 1mm, in the midline, at a rate of 0.2 μL per minute using a microinjector (UMP Pump II, World Precision Instruments). Twelve animals were used, with 6 animals per group. The 2 groups consisted of those transplanted with canine OECs transduced with pRRL-CMV-GFP (OECs group) and those transplanted with canine OECs transduced with pRRL-CMV-ChABC-SFFV-GFP (OECs+ChABC group). The number of animals used for quantitative analyses are detailed in the respective sections.

### Corticospinal axon labelling

Two weeks prior to termination of the experiment, descending CST axons were labelled by injecting 1μL of 10% biotinylated dextran amine (BDA) (MW 10 000, Molecular Probes) bilaterally into the sensorimotor cortex at 6 sites per hemisphere. All animals were anaesthetized as for the SCI / cell transplantation procedure and stereotaxic injections were made at a depth of 2 mm dorsoventrally into the sensorimotor cortex region using the following injection coordinates as determined from a microstimulation mapping study [40]: in reference to bregma; AP, anterior–posterior; L, lateral) AP −1.5 mm, L 2.5 mm; AP −0.5 mm, L 3.5 mm; AP +0.5 mm, L 3.5 mm; AP +1.0 mm, L 1.5 mm; AP +1.5 mm, L 2.5 mm; AP +2.0 mm, L 3.5 mm.

### Histopathology

Four weeks after SCI and cell transplantation, all animals were deeply anaesthetised using sodium pentobarbital (200mg/kg intraperitoneal) and transcardially perfused with 0.9% saline followed by 4% paraformaldehyde in 0.1M phosphate buffer. Tissue was collected immediately, mounted in 10% gelatin blocks and fixed overnight in 4% paraformaldehyde at 4°C, followed by immersion in 30% sucrose in phosphate buffer for 2 days at 4°C. Gelatin mounted tissue was frozen in optimized cutting compound (OCT, TissueTek) before sectioning. For ChABC activity, lesion size and cell survival determination and quantification of labelled axons, the spinal cord containing the lesion site was sectioned dorsally (40μm) with a cryotome and kept free-floating in phosphate buffered saline at 4°C for immunohistochemistry. 30μm transverse sections of the spinal cord at 2cm rostral and 2cm caudal to the injury were also obtained for determination of BDA labelled axons and PKCγ detection.

All primary antibodies were incubated overnight at 4°C. To verify complete disruption of the CST, transverse sections from each animal were immunostained with rabbit polyclonal anti-PKCγ (Santa Cruz, 1:1000), to visualise the CST, and CST appearance was compared rostral and caudal to the injury. To demonstrate *in vivo* ChABC activity, dorsal sections at the site of injury were immunostained with either mouse monoclonal anti-chondroitin-4-sulphate (MP Biomedicals, 1:5000), to reveal digested proteoglycan stub regions, or mouse monoclonal anti-neurocan (Developmental Studies Hybridoma Bank, concentrated version 1:50) to label intact CSPG. Complementary secondary antibodies were biotinylated goat anti-mouse or anti-rabbit (Vector Labs, 1:500). Sections were then processed using the avidin-biotin amplification method with conjugated peroxidase (Vector Labs, Vectastain ABC Elite Kit) and visualized with 3–3’-diaminobenzidine (DAB) and NiCl_2_ (DAB peroxidase substrate kit, Vector Labs). To identify transplanted cells, sections were immunostained using the following primary antibodies: mouse anti-nerve growth factor receptor (p75^NGFR^) (MAB5264, Millipore, Germany; 1:200), rabbit polyclonal anti-glial fibrillary acidic protein (anti-GFAP) to label astrocytes (Dako, 1:1000) and chicken anti-GFP (Abcam, 1:2000). Complementary secondary antibodies applied for 2 hours at room temperature included: donkey anti-mouse Alexa Fluor 546 (Invitrogen, 1:500), donkey anti-rabbit Alexa Fluor 660 (1:500, Invitrogen) and donkey anti-chicken Alex Fluor 488 (Invitrogen, 1:500). Fluorescently labeling of BDA axons was performed using an Avidin-Neutravidin Texas Red secondary (Thermofisher, 1:500) overnight at 4°C.

For determination of lesion size, dorsal sections were mounted and dried onto slides. Sections were stained for 1 min in Thionin, rinsed with distilled water, then dipped in 0.5% Eosin for 10 s, followed by rinsing with distilled water. Sections were dehydrated, mounted in xylene and the lesion size was quantified using ImageJ software (NIH, Bethesda).

### Quantification of corticospinal axons

Three serial dorsal sections incorporating the CST, from 8 animals (4 per group), were used for quantification of axonal sprouting, axonal regeneration and axonal dieback. Methods for sprouting and dieback were taken from Garcia-Alias, Barkhuysen (15) and Wang, Ichiyama (16). For axonal sprouting, each labelled CST axon sending a branch medially or laterally was counted. For axonal dieback, the number of labelled axons within 2mm of the rostral aspect of the lesion were counted. For axonal regeneration, the number of axons at 1mm, 2mm and 3mm caudal to the lesion were counted. Each axon count was normalized to the total number of labelled CST axons for each animal. This was determined by calculating the mean number of labelled axons in the 3 rostral transverse spinal cord sections. Sprouting axons were counted manually and all other counts were counted using a semi-automated method [41].

### Statistical analysis

Data are shown as mean +/- SEM. Axon count data were analysed by the Student’s *t* test where there were two groups and mixed ANOVA and Bonferroni *post hoc* analysis where each animal was split into 3 anatomical regions.

## Results

### Preparation of the canine olfactory ensheathing cell transplant

**Fig 1:**
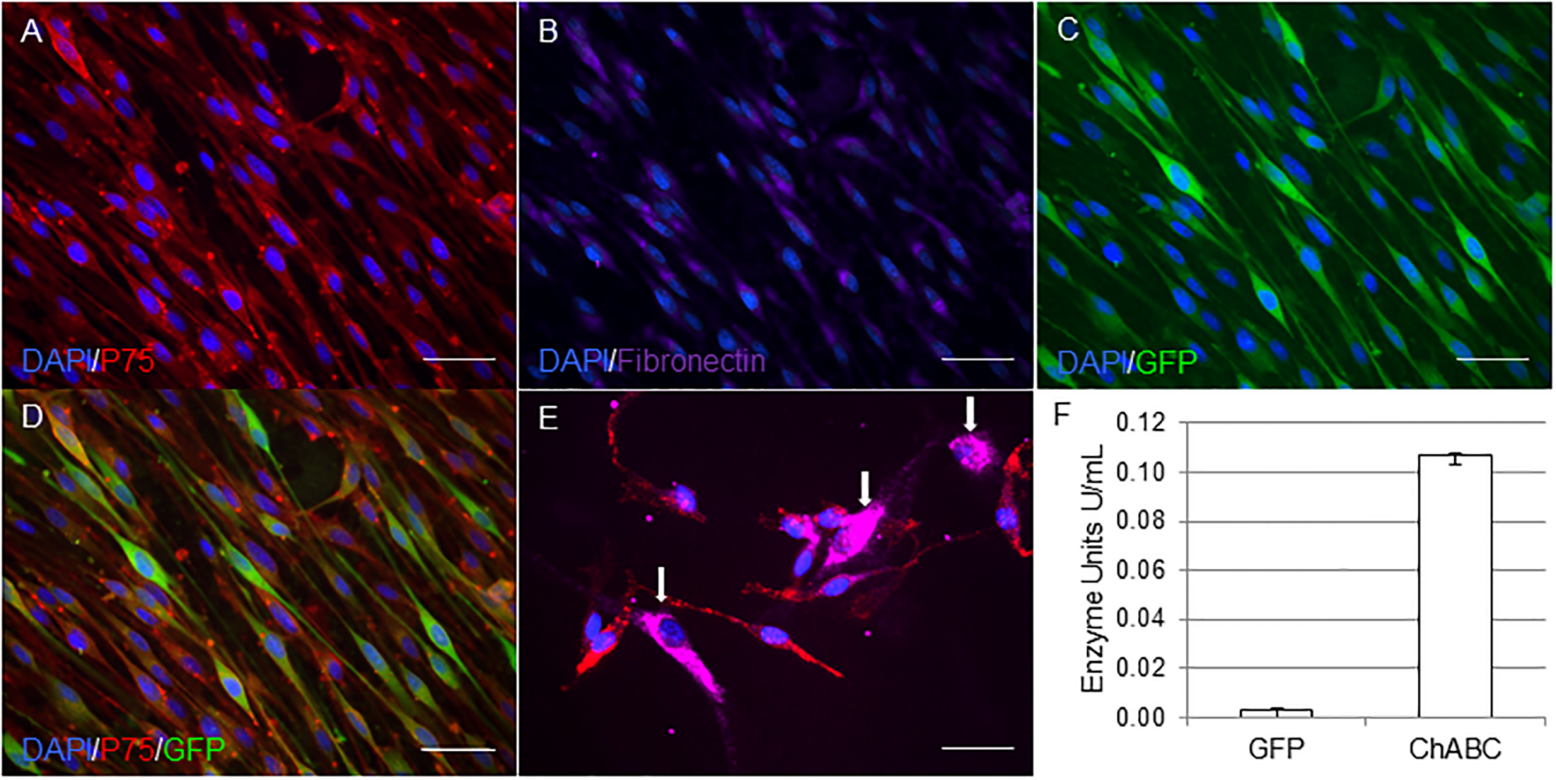
Immunohistochemical and biochemical analyses of canine mucosa OECs prior to transplantation. The cells have been genetically modified using the lentiviral vector pRRL-CMV-ChABC-SFFV-GFP to produce ChABC and GFP. All cells strongly expressed p75^NGFR^ (A) and weakly expressed fibronectin (B). 95% of OECs were transduced by the lentiviral vectors, as identified by the expression of GFP (C) and p75^NGFR^ (D). (E) Example of positive staining of fibronectin in contaminating fibroblasts (indicated by white arrows) in a different culture preparation. (F) Transduced cells secreted robust quantities of active ChABC in culture, as detected by the Morgan-Elson enzyme assay. Scale bar 50μm.

The purity of olfactory ensheathing cell cultures generated from the canine olfactory mucosa can vary from culture to culture, depending on the biopsy. We characterised the preparation used in this study by assessing the proportion of p75^NGFR^ positive cells and showed that the canine olfactory mucosa culture consisted of 100% p75^NGFR^ positive cells, with an elongated bipolar morphology, consistent with OEC phenotype (Fig 1A). These cells also expressed low levels of fibronectin, a property of OECs in culture as previously described [42]. No visible contaminating fibroblasts that are identified by robust fibronectin labelling and a round, flat morphology, as exemplified by a different culture containing fibroblasts in Fig 1E, were detected (Fig 1B). Using the transduction protocol described, 95% of cells were transduced at a multiplicity of infection of 10 (Fig 1C). Immediately prior to transplantation, cells transduced with the pRRL-CMV-ChABC-SFFV-GFP vector were secreting 0.1 U/mL of active ChABC *in vitro* and were easily identifiable by robust GFP expression. As expected, ChABC production by cells transduced with the pRRL-CMV-GFP vector were negligible (0.003±0.00U/mL) (Fig 1F). Enzyme production was not recorded at zero due to a small degree of error in the Morgan-Elson assay, and is highly unlikely to be due to the presence of ChABC given the make up of the vector construct.

### Canine olfactory ensheathing cells survive and migrate following transplantation into the injured spinal cord

**Fig 2:**
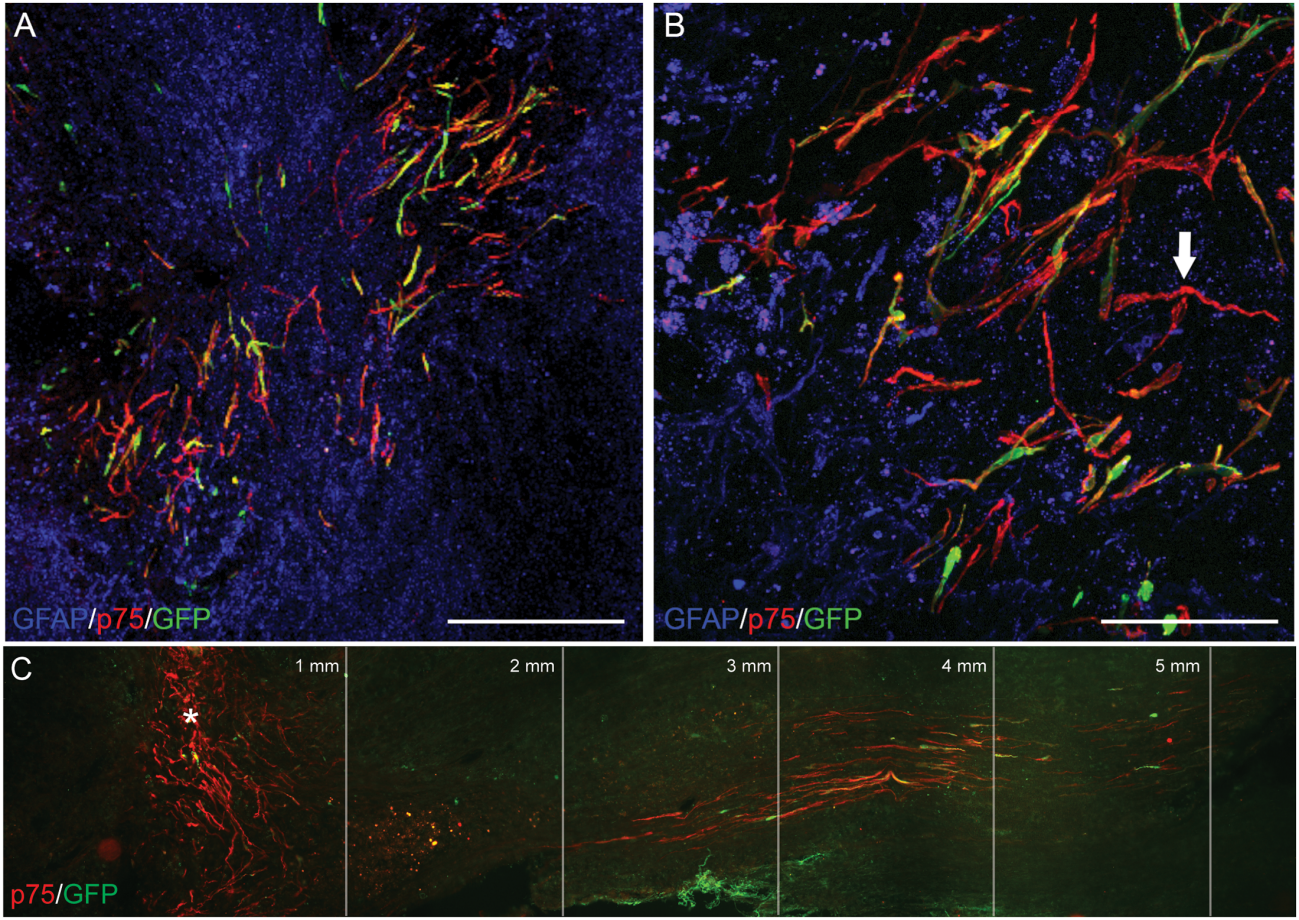
Transplanted canine mucosa OECs can be found at the lesion site at 4 weeks following transplantation. Genetically modified OECs can be identified due to the expression of GFP (green). These bipolar spindle shaped cells also express the nerve growth factor receptor p75^NGFR^ (red, white arrow). Cells intermingle with reactive astrocytes expressing GFAP (blue) around the lesion penumbra (A, B). Elongated OECs were seen migrating up to 5mm caudal to the lesion within the dorsal column white matter tracts (C). * = lesion centre. Scale bar A, 100μm. B, 25μm.

Following culture and characterisation of OECs, 80,000 of either GFP- or ChABC-producing OECs were transplanted in a total volume of 2μL rostral and caudal to the spinal cord lesion. Transplanted OECs expressing GFP were identified four weeks following transplantation into nude rat spinal cords (Fig 2). Transplanted cells were found intermingled with reactive astrocytes at the site of injury (Fig 2A). These cells continued to express p75^NGFR^ *in vivo* and maintained their bipolar morphology seen in culture (Fig 2B). The mean percentage of cells that survived to 4 weeks post-transplantation was 6.5% (+/- SEM, 2.5%), with no significant differences in survival between the two groups (10.4±4.0 for GFP cells vs 7.0 ± 0.9 for ChABC-producing cells). ChABC-producing OECs were found migrating up to 5mm caudal to the site of transplantation within white matter tracts (Fig 2C), with the majority of cells remaining at the site of spinal cord lesion. However, further assessment revealed that the number of cells migrating away from the injection site did not differ significantly between the OEC-GFP and the OEC-ChABC groups, possibly due to the small sample size.

### Olfactory ensheathing cells secreting chondroitinase ABC digest chondroitin sulphate proteoglycans *in vivo*

**Fig 3:**
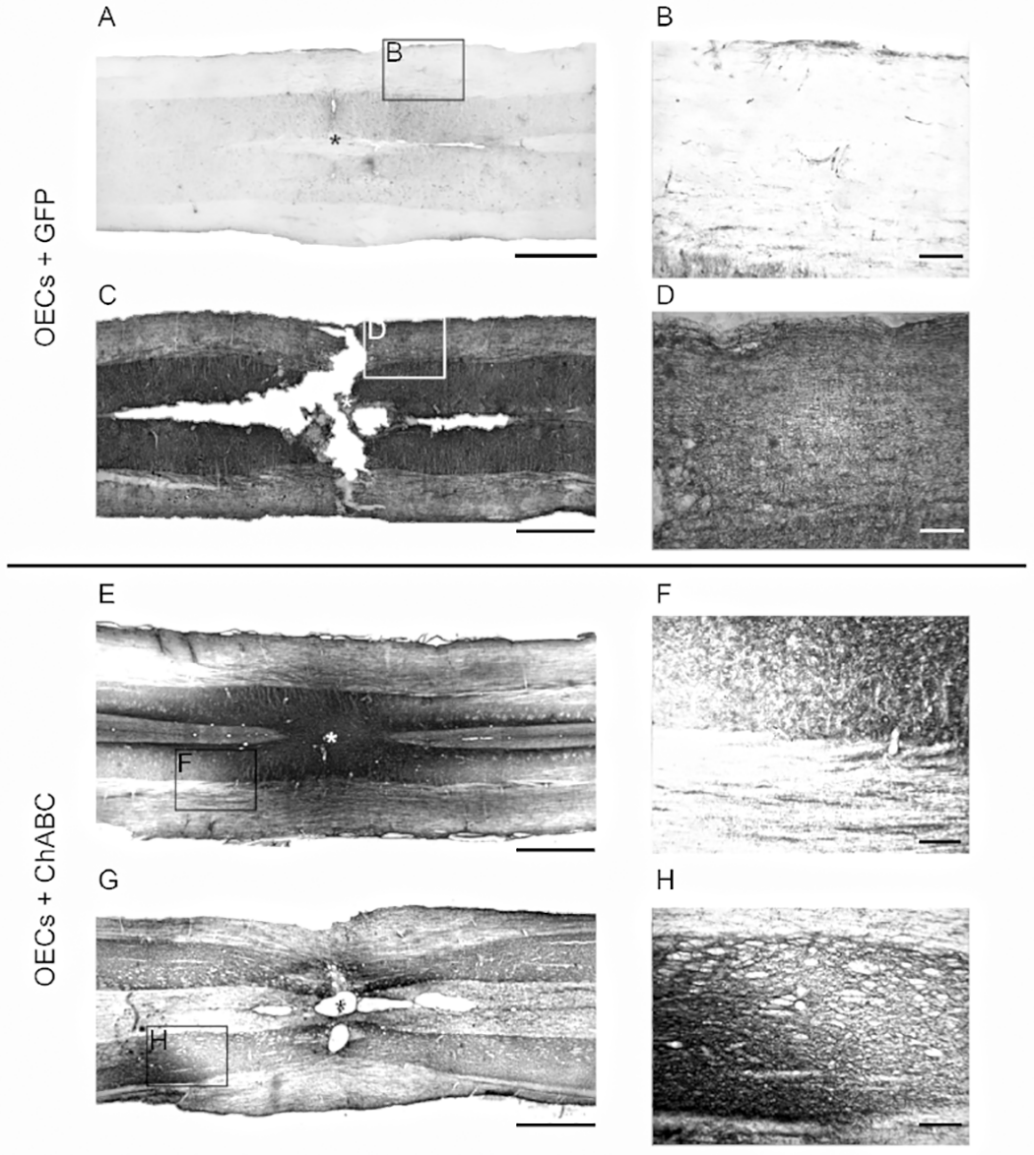
CSPG digestion by canine mucosa OECs producing ChABC *in vivo*. OECs expressing GFP did not digest CSPGs as shown by the lack of C4S immunolabelling in (A) and the presence of robust neurocan immunolabelling in (C). (B) and (D) are high magnification images of the regions highlighted in (A) and (C). OECs producing ChABC digested CSPGs revealing C4S epitopes around the site of injury (E). High magnification image of the highlighted region in E demonstrates the transition between digested (top right of F) and intact (bottom left of F) CSPG (F). CSPG digestion removes neurocan from the site of injury and in a 3mm halo around the lesion (G). High magnification image of the highlighted region in G demonstrates the absence of neurocan immunolabelling (right of box H) around the lesion. Asterisk * on panel A, C, E and G represents the experimental lesion. Scale bar A, C, E, G 1mm. Scale bar B, D, F, H 200μm.

The spinal cord sections were examined for evidence of CSPG digestion by transplanted OECs secreting ChABC, compared to GFP-expressing OECs (Fig 3). The antibody anti-C4S detects digested glycosaminoglycan stubs which are only present following ChABC-mediated digestion of CSPG. Spinal cord sections including the lesion site showed no labelling in all animals that received GFP-expressing OECs (Fig 3A and 3B). Furthermore, widespread labelling for intact CSPG was present, as detected by neurocan immunolabelling (Fig 3C and 3D). In contrast, spinal cord sections from animals transplanted with ChABC-producing OECs demonstrated C4S labelling up to 3mm surrounding the lesion, providing evidence of functional ChABC activity (Fig 3E). High magnification at the border of CSPG digestion showed the clear distinction in the presence and absence of C4S labelling (Fig 3F). Intact CSPG labelled with the anti-neurocan antibody also demonstrated a clear halo of digested CSPG surrounding the lesion (Fig 3G and 3H).

**Fig 4:**
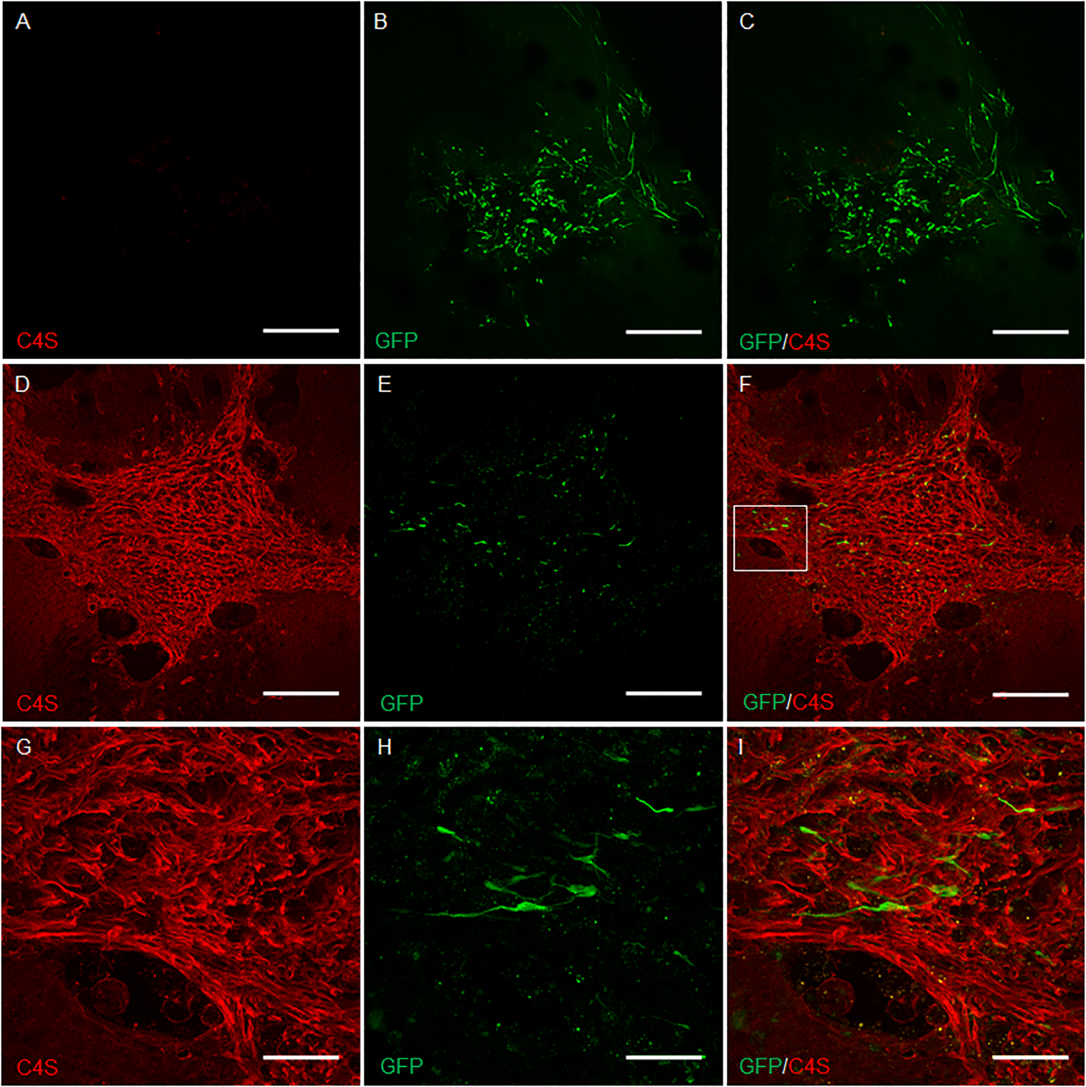
Canine mucosa OECs producing ChABC digest CSPGs at the site of spinal cord lesion. OECs lacking ChABC do not cause any visible CSPG digestion as shown by the lack of C4S immunoreactivity surrounding OECs expressing GFP (A-C). There is widespread C4S immunolabelling in animals transplanted with ChABC-producing OECs, particularly around the site of the spinal cord lesion (D-F). High magnification image of box in (F) shows easily identifiable GFP labelled transplanted OECs associated with regions of intense CSPG digestion (G-I). Scale bar A, B, 100μm C, 25μm.

Spinal cord sections were examined for the presence of fluorescently labelled OECs at the lesion site and the presence or absence of C4S labelling surrounding cell transplants (Fig 4). C4S labelling at the site of transplantation was absent in all animals that received OECs producing GFP (Fig 4A–4C). All animals transplanted with ChABC producing OECs showed C4S labelling surrounding transplanted cells (Fig 4D–4F). The most intense C4S labelling was localised to the site of SCI, likely due to the higher quantity of CSPGs present within the glial scar. A lower intensity of C4S labelling was also present surrounding these intensely labelled regions, possibly due to digestion of CSPGs in perineuronal nets. High magnification imaging of this C4S intense region showed transplanted cells, identified by robust GFP expression and a bipolar spindle like morphology (Fig 4G–4I).

### Chondroitinase ABC producing olfactory ensheathing cells increase axonal sprouting following corticospinal tract injury

**Fig 5:**
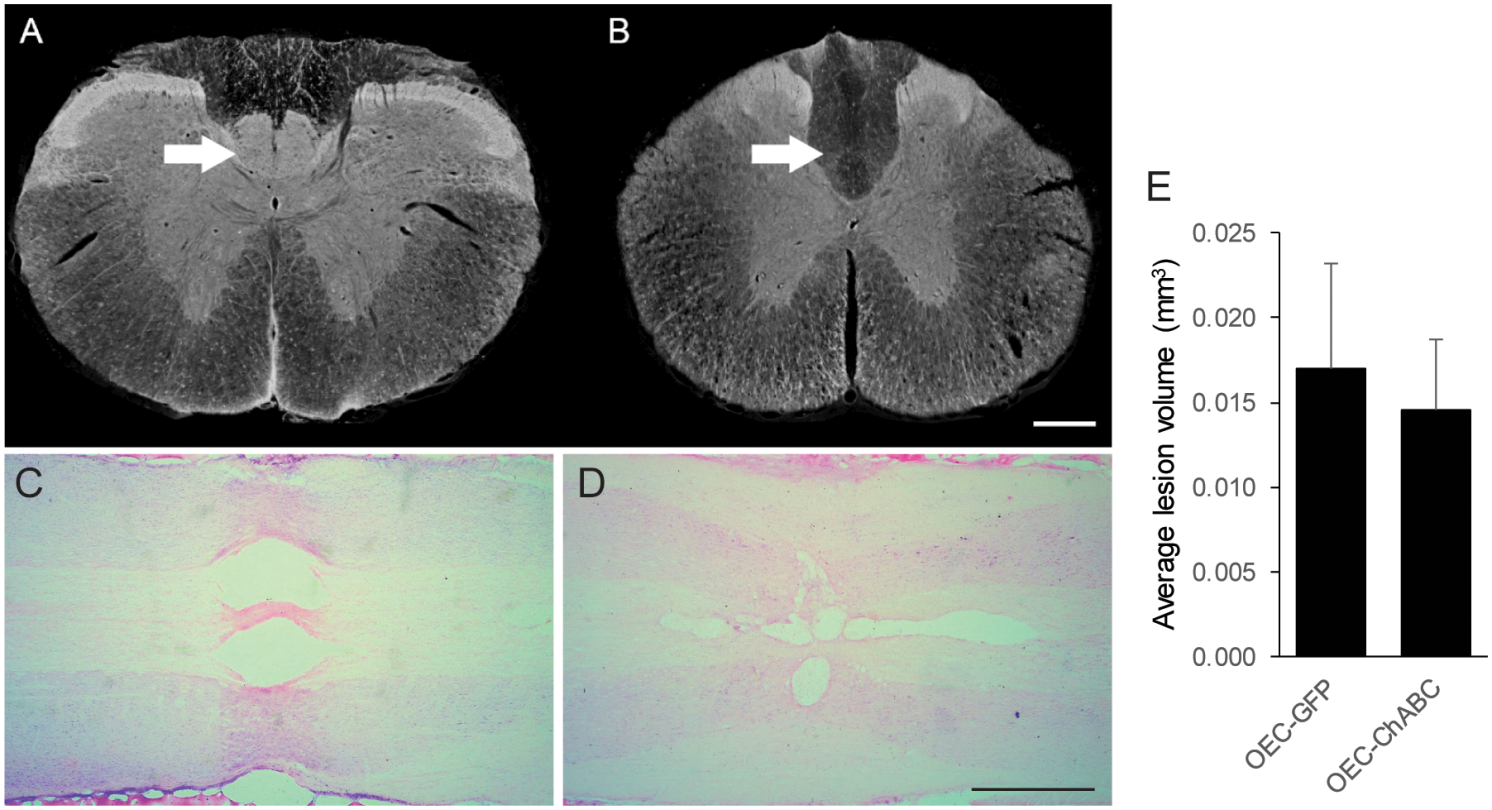
Demonstration of complete CST injury. Transverse section of the cervical spinal cord rostral to the dorsal column crush injury immunolabelled for PKCγ to show intact CST axons (A). The arrow indicates the location of the CST in the dorsal columns. Transverse section taken caudal to the site of injury showing the absence of corticospinal axons in the dorsal columns (B). Spinal cord lesion can be visualised in thionin-eosin stained spinal cords of animals receiving OEC-GFP (C) and OEC-ChABC (D). No significant differences in the lesion size were detected between the two groups (n = 3). Scale bar 500μm.

Corticospinal tract (CST) axons were labelled by injecting 10,000 MW biotinylated dextran amine (BDA) bilaterally into the sensorimotor cortex. All 12 animals had complete destruction of the CST at the C4 spinal cord segment following a dorsal column crush injury, as shown by the absence of PKCγ labelling caudal to the site of injury (arrowhead Fig 5B, S1 Fig). The CST can be seen rostral to the lesion in the ventral aspect of the dorsal funiculus (arrowhead Fig 5A). Histological examination of the H&E stained sections showed that the lesion size did not significantly differ between the two groups (Fig 5C–5F).

**Fig 6:**
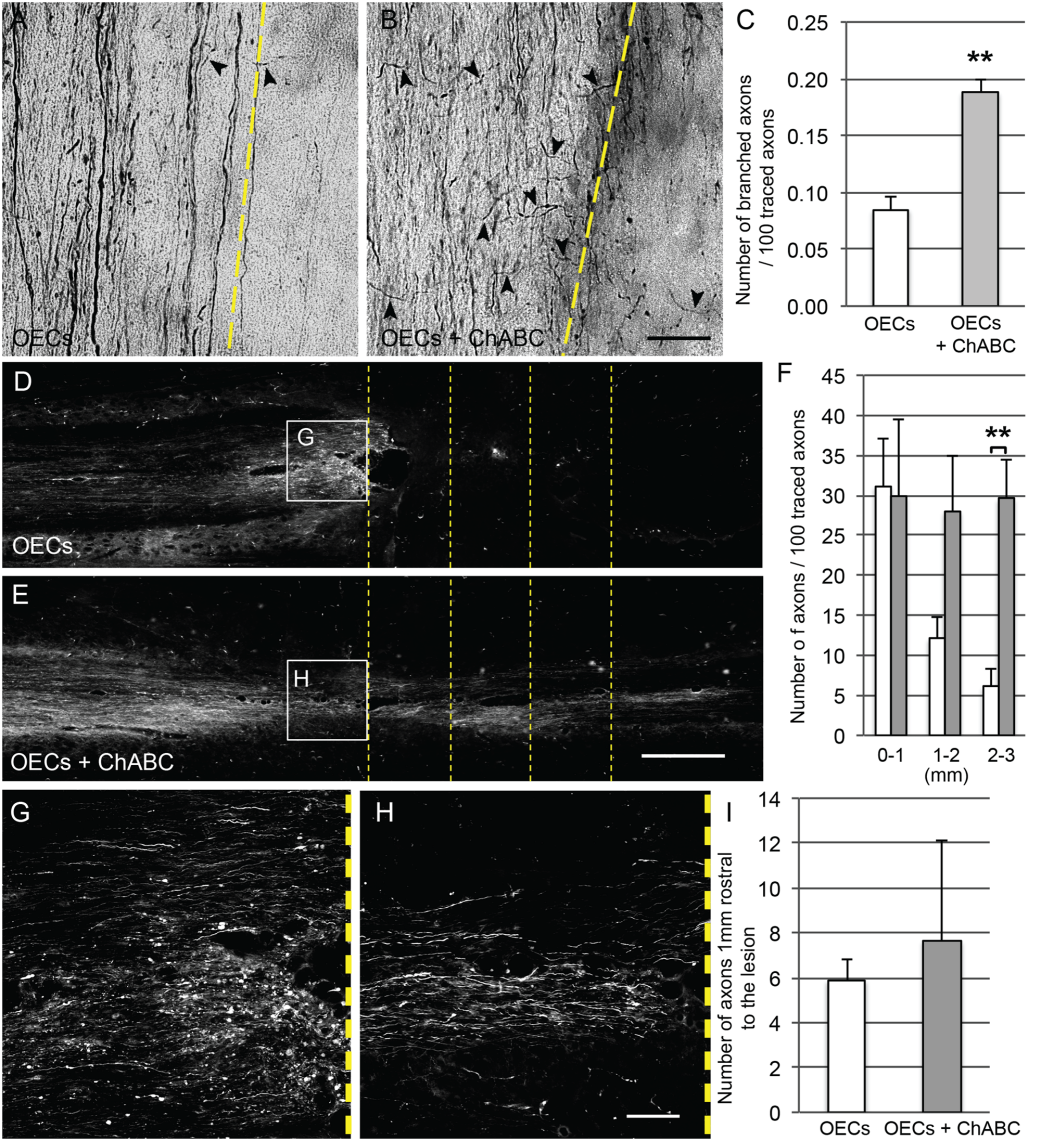
ChABC produced by genetically modified OECs leads to increased sprouting of CST axons rostral to the lesion and increased number of CST axons caudal to the lesion. Sprouting CST axons were observed rostral to the lesion, as indicated by arrowheads (A, B). The yellow dashed line in A and B indicates the grey to white matter boundary and white matter is to the left of the images. Significantly more sprouting CST axons were seen in animals transplanted with OECs producing ChABC (C; P = 0.0028, Student’s *t*-test, n = 4). BDA-labelled axons travelling from rostral (left) to caudal (right) in the dorsal CST are shown in white (D, E). Axons were seen extending over 3mm caudal to the lesion in all animals but significantly more axons were seen in animals transplanted with OECs producing ChABC (F, n = 4). The degree of axonal dieback, assessed by counting the number of CST axons immediately rostral to the lesion, was not different between groups (G, H, I, n = 4). High magnification images G and H are taken from images D and E with the dotted line to the right of the images indicating the lesion. ** = p<0.01. Scale bar A, B = 50μm. D, E = 1mm. G, H = 0.25mm.

The number of branching axons arising rostral to the damaged CST was compared between the two transplant groups. The count was normalised for each animal based on the number of BDA labelled CST axons identified in a rostral cervical transverse section. Branching axons were seen in both groups but there was a significant increase in the number of branching axons in animals transplanted with ChABC producing OECs (Fig 6C). Occasionally these branching axons crossed the grey to white matter boundary (Fig 6A and 6B).

The number of intact axons identified caudal to the lesion was compared between groups (Fig 6D, 6E and 6F). Within 1mm caudal to the lesion there was no difference in the number of axons between spinal cord lesions containing control OECs and OECs producing ChABC. At 1-2mm caudal to the lesion there was a higher number of axons seen in animals transplanted with OECs producing ChABC however this did not reach significance. At 2-3mm caudal to the lesion there were significantly more CST axons seen in animals transplanted with OECs producing ChABC compared to those receiving OEC-GFP transplants (p = 0.005, mixed ANOVA). These axons seen caudal to the lesion could either have sprouted from the unlesioned lateral CST or represent regenerating axons. In animals transplanted with OECs producing ChABC, tortuous axons were seen traversing the injury, closely associated with the lesion cavity, and extending into the normal portion of spinal cord caudally. In contrast, this was not seen in animals transplanted with GFP expressing OECs, where axons ended at the lesion in dystrophic endbulbs (S2 Fig).

As the cell transplant was given at the time of spinal cord section (i.e. mimicking the acute phase of SCI), it may have provided some neuroprotection to damaged neurons. To assess this we compared the degree of axonal dieback between the two groups (Fig 6G, 6H and 6I). No significant difference was observed between the two groups and the axon counts were similar between groups. BDA-labelled CST axons could be seen abutting the cystic cavity in animals transplanted with control OECs and OECs producing ChABC.

## Discussion

In this proof of concept study, we have shown that OECs dissected from the canine mucosa and cultured *in vitro* can be genetically modified to secrete functional mammalian-modified ChABC *in vivo* at the site of SCI following transplantation. This led to digestion of CSPGs in and around the site of injury and was associated with an increase in axonal sprouting and putative axonal regeneration in the CST.

The OEC cultures used for these experiments were collected from the olfactory mucosa of a companion dog immediately following euthanasia using a minimally invasive technique. The purity of OEC cultures can vary from culture to culture if no purification steps are employed, and this has been documented in samples of the olfactory mucosa from humans [43, 44]. For clinical translation, characterisation of cell purity and identification of any contaminating cells prior to transplant will be crucial in light of a case report of a spinal cord mass that developed after olfactory mucosal cell transplantation in a patient with a spinal cord injury [45]. The mass comprised cells of the respiratory epithelium and submucosal glands, however it is noteworthy that this transplant was neither characterised nor cultured prior to transplantation. In other studies reporting long-term follow-up of humans transplanted with ‘cultured OECs’, no adverse effect was observed [46–47]. Protocols are now available for culture of human OECs in view of transplantation [48]. In our transplantation experiments, we did not find any contaminating fibroblasts, and all cells were p75^NGFR^ positive, a marker whose utility for OEC purification has been recently highlighted [49]. Previous cultures using this technique have documented cell purities of around 85% [37], and the high percentage of p75^NGFR^ positive cells, presumed to be olfactory ensheathing cells, may be an important contributing factor to facilitating axonal regeneration. As the p75^NGFR^ marker also detects Schwann cells, it is possible that our culture contains Schwann cells but this is unlikely given that OECs are the predominant p75^NGFR^ positive cell type in the olfactory mucosa and we and others practice careful dissection and removal of respiratory mucosa and blood vessels (the main source of Schwann cells) before the start of the culture. Nonetheless, in the absence of a specific marker for OECs we cannot discount the possibility that contaminants are present in OEC transplants.

We chose to explore this *ex vivo* gene delivery method as it can provide a further degree of safety compared with *in vivo* gene delivery [50]. OECs, a fully differentiated cell type, were transduced *in vitro* using a self-inactivating lentiviral vector, prior to transplantation, with no exposure of viral vector to host tissue. Meta-analysis of 49 OEC transplantation studies in rat models of SCI by Watzlawick et al. [51] indicated that the stable effect sizes range between 10,000–100,000 cells in a volume of up to 2.9μL, with high dose cellular transplants in high injection volumes causing neurotoxicity. Based on these and previous studies [30], we transplanted 80,000 cells in a total volume of 2μL rostral and caudal to the spinal cord lesion. Transplantation of the genetically modified OECs provided a sustainable source of ChABC to effectively digest the inhibitory CSPGs at the lesion site. Other groups have investigated the safety of long-term delivery of ChABC to the CNS [23, 52–54] and found no evidence of neuropathic pain or autonomic dysreflexia, both theoretical complications of inducing plasticity in host axons [55–57]. A further desirable safety feature would be to regulate expression of ChABC, for example by using a tetracycline regulatable system [58, 59] whereby ChABC production could be controlled by systemic tetracycline analogues. This could allow the gene therapy to be switched off at a precise clinical end point or if adverse events are identified, further improving the safety of the therapy. Another advantage of *ex vivo* gene delivery is that it allows tracking of transplanted cells, in experimental injury models, by incorporating a fluorescent reporter gene [60].

Intense anti-C4S labelling was found at the site of injury in animals transplanted with OECs producing ChABC. The C4S antibody recognises a glycosaminoglycan stub that is only revealed following digestion of CSPGs with ChABC. The intense labelling is likely due to digestion following the up-regulation of CSPGs that occurs at the glial scar [61–63]. The observation of strong anti-C4S labelling surrounding this CSPG rich region is also likely due to digestion of CSPGs associated with the perineuronal nets of normal neuronal synapses [64]. These two distinct sites containing intense staining for digested CSPGs are consistent with the two main mechanisms of action of ChABC following SCI, namely removal of CSPGs from the glial scar promoting axonal regeneration and the breakdown of perineuronal nets promoting plasticity of surviving neurons [8, 65].

More axons were observed caudal to the injury site in animals transplanted with OECs producing ChABC compared to controls and this difference was most evident at 2 to 3mm caudal to the lesion. This suggests that the combination of OECs with ChABC enhanced the growth-promoting properties of the OECs, enabling longer-range regeneration of injured or spared axons. Some of these axons could be seen traversing the injury site and extending caudally; these may be regenerating axons or plasticity elicited from spared fibres. It is also possible that axons may have bypassed the lesion and reconnected with the caudal CST, a recognised finding in spinal cord injured animals treated with ChABC [16, 66].

We observed a mean survival rate of 6% at 4 weeks post-transplantation. Poor cell survival may be due to a number of factors, such as the timing of the transplantation, immune rejection of the grafted cells particularly in a xenotransplant paradigm, hostile environment of the spinal cord lesion or shock to the cells from culture to transplant [67]. Watzlawick *et al*. [51] in a meta-analysis study suggested that transplantation in the hyperacute phase (5–30 min) was associated with greater efficacy than transplantation in the subacute or chronic phases. Poor cell survival is possibly due to the xenotransplantation of canine cells into the rodent species. Although athymic rats that are T-cell deficient were used, these animals have normal B-cell function and increased Natural Killer (NK) and macrophage cell populations. There is some evidence that other components of the immune system can contribute to xenogeneic rejection, for example NK cells play a more prominent role in xenograft rejection [68–69]. Khankan *et al*. [70] showed that 14±8% OECs survive at the spinal cord lesion at 8 weeks post-transplantation in immunosuppressed rats (cyclosporine A) receiving an allograft. Therefore, the survival rate of our transplanted cells is not surprising. Nonetheless, it will be necessary to test this approach in a chronic injury model. We observed ChABC-producing OECs migrating up to 5mm from the site of transplantation, however upon further analysis there were no significant differences in the migration rate between the two groups, possibly due to small sample size and therefore further investigation is warranted. OEC migration is a common observation in SCI studies [71–74] and the degree of migration is thought to have an influence on functional recovery [75, 76]. OECs are thought to migrate ahead of regenerating axons and encourage axon growth by secreting neurotrophic factors [77]. Recently it has been demonstrated that CSPGs can impair OEC migration, and that addition of ChABC increased OEC migration over CSPG substrates in an in vitro migration assay [78].

In previous spinal cord OEC transplantation studies in companion dogs, some cases ‘responded’ to the therapy whereas others were ‘non-responders’ and this has highlighted the need to further improve the OEC transplantation therapy [30]. To achieve this, we have undertaken a ‘proof-of-concept’ study to test canine OECs genetically modified to secrete ChABC in the context of a laboratory using homogeneous animals with similar injuries. This can quickly detect the efficacy of the proposed treatment before embarking in a more complex clinical trial in companion dogs (usually requiring at least 3 years). We used a xenotransplant model (canine cells into rodents), and athymic rats to reduce the risk of cell transplant rejection. Transplanted cells survived at least 4 weeks. Cell survival times are likely to be shorter in immunocompetent animals but studies utilising autologous transplantation, in dogs and people, have indicated that transplant rejection is less likely to be an issue in clinical cases [30, 32]. Having now gained knowledge on the potential of engineered canine ChABC OECs, the next logical step would be to determine the longer term survival of these cells and functional recovery in a chronic injury model. This could lead to further testing in companion dogs used as a translational model, as recently highlighted by the CANSORT-SCI group [36].

## Conclusions

The current study shows that canine OECs, genetically modified to produce ChABC, are capable of digesting CSPGs following transplantation into the injured spinal cord and that this CSPG digestion is associated with significantly more CST axons caudal to the injury, possibly due to increased plasticity or new growth of axons. This provides an important proof-of-concept and positions OECs expressing ChABC as a viable method to deliver ChABC inconjunction with the well-known added benefits of OEC transplantation. An efficacy question remains but we are now well positioned to take this therapy further, for example by taking advantage of companion dogs spontaneously affected by SCI. These are available to neuroscientists through large veterinary hospitals to take part in randomised controlled clinical trials that could allow detection of real-life functional benefits.

## Supporting information

S1 Fig Demonstration of complete CST injury. Transverse section of the cervical spinal cord caudal to the dorsal column crush injury immunolabelled for PKCγ to show absence of corticospinal axons in the dorsal columns in all animals, indicating complete CST injury.
(TIF)

S2 Fig BDA labelled corticospinal axons at the site of injury. **Tortuous axons (arrowhead in A) can be seen traversing the site of injury and extending caudally (A).** Axons are closely associated with the lesion cavity and contain swellings suggestive of active growth (arrowhead in B) (B). In control animals transplanted with OECs alone, axons end abruptly in retraction bulbs suggestive of abortive regeneration (C). * = lesion centre. Caudal is to the right of the images. Scale bar 100μm.
(TIF)
