# Supplementary figures and images for: Transplantation of canine olfactory ensheathing cells producing chondroitinase ABC promotes chondroitin sulphate proteoglycan digestion and axonal sprouting following spinal cord injury
Source: PLoS One. 2017 Dec 11;12(12):e0188967. doi: 10.1371/journal.pone.0188967 (PMC5724818; doi:10.1371/journal.pone.0188967)

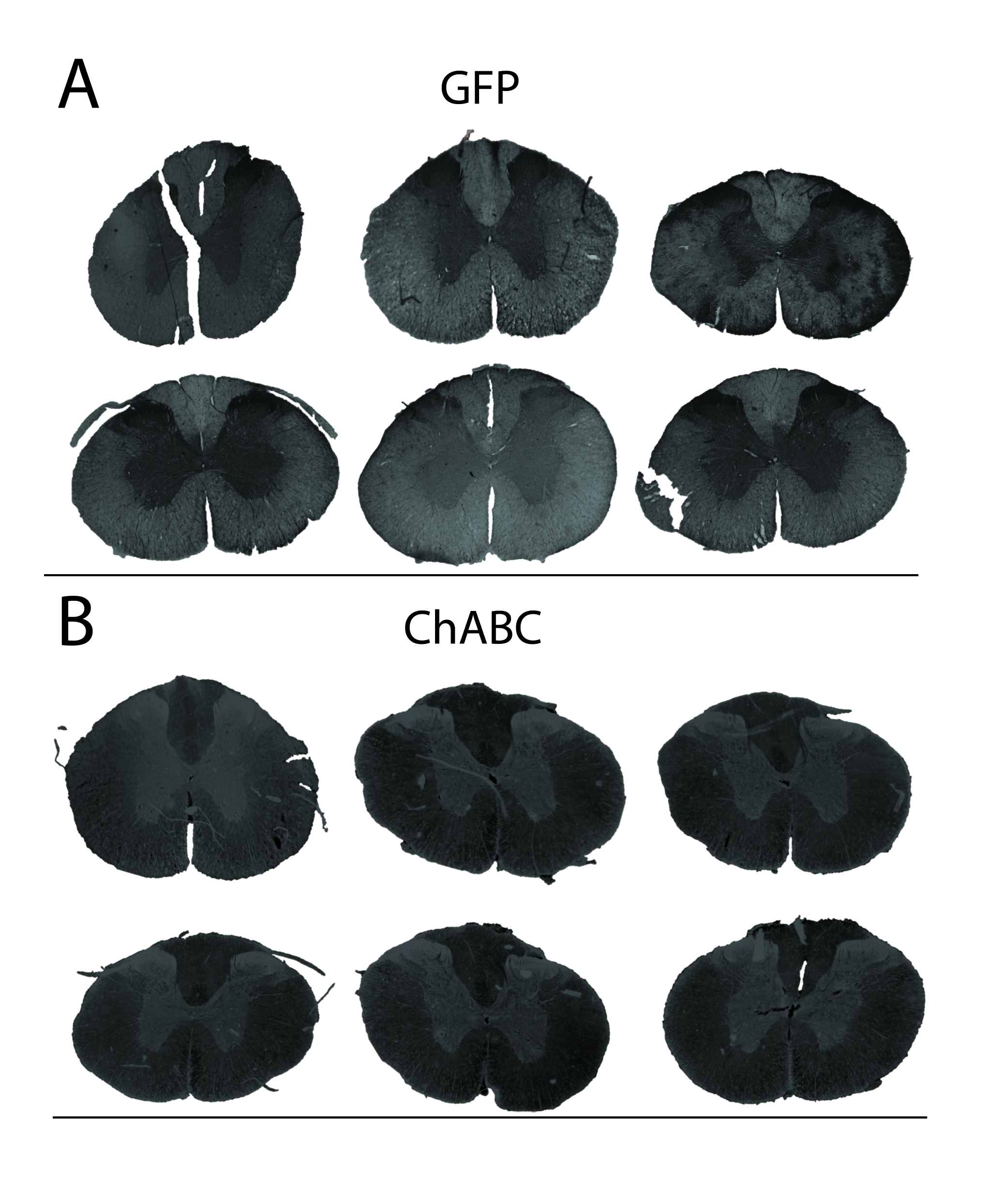

Supplement: S1 Fig — Transverse section of the cervical spinal cord caudal to the dorsal column crush injury immunolabelled for PKCγ to show absence of corticospinal axons in the dorsal columns in all animals, indicating complete CST injury. (TIF) [file pone.0188967.s001.tif]
